# Improved Coronary Artery Visualization Using Virtual Monoenergetic Imaging from Dual-Layer Spectral Detector CT Angiography

**DOI:** 10.3390/diagnostics13162675

**Published:** 2023-08-14

**Authors:** Tommaso D’Angelo, Ludovica R. M. Lanzafame, Antonino Micari, Alfredo Blandino, Ibrahim Yel, Vitali Koch, Leon D. Gruenewald, Thomas J. Vogl, Christian Booz, Giuseppe M. Bucolo, Maria Teresa Cannizzaro, Giorgio Ascenti, Silvio Mazziotti

**Affiliations:** 1 Diagnostic and Interventional Radiology Unit, BIOMORF Department, University Hospital Messina, 98124 Messina, Italy; ludovicalanzafame@gmail.com (L.R.M.L.); ablandino@unime.it (A.B.); giuseppebucolo94@gmail.com (G.M.B.); gascenti@unime.it (G.A.); smazziotti@unime.it (S.M.); 2 Department of Radiology and Nuclear Medicine, Erasmus MC, 3015 GD Rotterdam, The Netherlands; 3 Department of Clinical and Experimental Medicine, DIMED, University Hospital Messina, 98124 Messina, Italy; antoniomicari96@gmail.com; 4 Division of Experimental Imaging, Department of Diagnostic and Interventional Radiology, University Hospital Frankfurt, 60590 Frankfurt am Main, Germany; ibrahim.yel@kgu.de (I.Y.); vitali.koch@kgu.de (V.K.); leondavid.gruenewald@kgu.de (L.D.G.); thomas.vogl@kgu.de (T.J.V.); boozchristian@gmail.com (C.B.); 5 Radiology Unit (CAST), University Hospital Catania, “Policlinico G. Rodolico–San Marco”, 95123 Catania, Italy; maraterex@yahoo.it

**Keywords:** computed tomography angiography, coronary artery disease, coronary stenosis, dual-energy computed tomography

## Abstract

**Background**: To evaluate if coronary CT angiography (CCTA) monoenergetic reconstructions, obtained with a dual-layer spectral detector computed tomography (DLCT) system, offer improved image quality compared with 120 kVp conventional images without affecting the quantitative assessment of coronary stenoses. **Methods**: Fifty CCTA datasets (30 men; mean age: 61.6 ± 12.3 years) acquired with a DLCT system were reconstructed using virtual monoenergetic images (VMI) from 40 to 100 keV with 10 keV increment and compared with conventional images. An analysis of objective image quality was performed, evaluating the signal- and contrast-to-noise ratio. For the subjective assessment, two readers used a 5-point Likert scoring system to evaluate sharpness, noise, demarcation of coronary plaques, vascular contrast, and an overall score. Furthermore, coronary stenoses were analyzed for each vessel to describe the diagnostic agreement between monoenergetic images and conventional images. **Results**: The objective image analysis showed that all reconstructions from 70 keV to 40 keV show higher SNR (from 61.33 ± 12.46 to 154.22 ± 42.91, respectively) and CNR (from 51.45 ± 11.19 to 135.63 ± 39.38, respectively) compared with conventional images (all *p* < 0.001). The 40 keV monoenergetic images obtained the best average score for sharpness, vascular contrast, and for the overall impression (all with *p* < 0.001). The detection and grading of stenoses of the coronary arteries with conventional and monoenergetic images at 70 keV and 40 keV showed an overall excellent interobserver agreement (k= 0.81 [0.72–0.91]). **Conclusions**: The 40 keV virtual monoenergetic images obtained with a DLCT system allow the objective and subjective image quality of coronary CT angiography to be improved.

## 1. Introduction

Coronary computed tomography angiography (CCTA) has become the preferred non-invasive test for the diagnostic workup of patients with low-moderate risk of coronary artery disease (CAD).

Thanks to its high sensitivity to detect and characterize coronary stenosis, its increased availability, and the speed and ease of execution, CCTA is becoming a widespread technique in radiologists’ clinical routine. 

Over the last two decades, the CCTA diagnostic value has been further expanded by the use of dual-energy computed tomography (DECT) applications [1].

Dual-energy CCTA (DE-CCTA) has been shown to provide additional information compared to conventional single-energy computed tomography [2,3,4,5,6]. Among the DECT main features, virtual monoenergetic imaging (VMI) at different kilo-electron volt (keV) levels has shown advantages both for unenhanced and contrast-enhanced CT. VMI at higher energy levels has been shown to reduce high-attenuation and blooming artifacts [7,8], whereas lower-keV monoenergetic images improve the luminal contrast of coronary arteries [9,10,11,12]. 

Recently, a novel dual-layer spectral detector CT (DLCT) system was released, which enables the simultaneous acquisition of low- and high-kV data from a single detector. Although the benefits derived from VMI reconstructions have already been investigated, current literature has mainly focused on VMI derived from dual-source or rapid-kV switching DECT systems [13,14]. Different from the aforementioned technologies, both dual-layer and the newest photon-counting CT platforms are based on spectral data directly derived from CT detectors. This mechanism would enable increased values of contrast-to-noise ratio (CNR) to be obtained while reducing the noise at low energy levels [15].

The aim of our study was to investigate whether VMI obtained from the DLCT platform improves objective and subjective image quality compared to 120 kVp CCTA conventional images, and to prove whether coronary stenosis quantification may be affected by using VMI.

## 2. Materials and Methods

This retrospective, monocentric study was approved by our institutional review board, and informed consent was obtained from all patients.

### 2.1. Study Population 

We reviewed our institutional database to identify consecutive patients who had undergone CCTA on the DLCT platform between June 2022 and December 2022.

Deviations from our DE-CCTA image acquisition or reconstruction protocol, as well as intravenous contrast injection protocol, led to patient’s exclusion. Only patients with a body mass index (BMI) between 18.5 and 35 kg/m^2^ were considered eligible. 

### 2.2. DECT Acquisition Protocol

All included CCTA scans were performed on a DLCT platform (IQon Spectral CT, Philips Healthcare, Eindhoven, The Netherlands).

Preliminarily, all patients received a premedication with a sublingual vasodilator agent (isosorbide dinitrate) if not contraindicated and a β-receptor blocker was administered to all patients with a heart rate (HR) ≥ 70 (esmolol hydrochloride injection, Baxter, Chicago, IL, USA).

Image acquisition was performed in the craniocaudal direction using inspiratory breath-hold. The study protocol consisted of an unenhanced scan for the assessment of coronary calcium score and a coronary angiographic phase performed after the intravenous administration of a non-ionic contrast agent (Iomeron 400 mgI/mL; Bracco, Milan, Italy) at 0.9 mL/kg bodyweight and a flow rate of 5 mL/s, followed by a 50 mL saline chaser. Automated bolus tracking was used by placing a region of interest (ROI) in the descending aorta at the level of the pulmonary artery with a 120 HU threshold level and a 6 s delay. 

Acquisition parameters were set as follows: tube voltage 120 kVp with automatic modulation of mAs; nominal layer thickness 0.67 mm; scan interval—0.34 mm; detector collimation—64 × 0.625 mm; tube rotation time—0.27 s; matrix 512 × 512; FOV 220 mm. The retrospective electrocardiographically triggered scan mode and the phase with less motion artifacts were used.

### 2.3. Image Reconstruction

All conventional CCTA datasets were reconstructed with the recommended cardiac reconstruction kernel (cardiac routine). Spectral reconstruction software (Spectral Recon, Philips Healthcare, Eindhoven, The Netherlands) was used to obtain spectral base images (SBI). Iterative model reconstruction (IMR Cardiac, Philips Healthcare) algorithm was used both for conventional and SBI datasets.

### 2.4. Objective Image Quality Analysis

Conventional and spectral datasets were evaluated using a dedicated software (IntelliSpace Portal Version 8.0, Philips Healthcare). VMI datasets were displayed at six different energy levels, ranging from 40 to 100 keV, with 10 keV increment.

Each dataset was evaluated by a radiologist with six years of experience in CCTA. ROIs were placed centrally in the aortic root, in the left main coronary artery, in the proximal segments of the left anterior descending, the circumflex and the right coronary artery, in the basal region of the interventricular septum, and in the subcutaneous fat. The ROI size was not smaller than 2 mm^2^.

Measures of the attenuation values (HU) and standard deviations (SD) were repeated twice, averaged, and compared for conventional and monoenergetic images.

For each dataset, we used the following equation to calculate CNR values of coronary vessels [16]:

CNR = (HUvessel − HUmyocardium)/SDfat

Signal-to-noise ratio (SNR) was calculated as follows:

SNR = HUvessel/SDfat

### 2.5. Subjective Image Assessment

**Figure 1:**
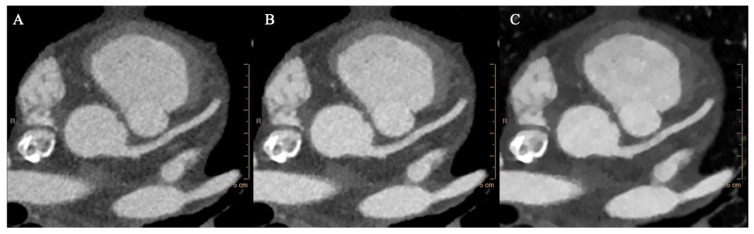
Conventional (**A**), 70 keV (**B**), and 40 keV (**C**) VMI axial images showing the aortic root at the level of the left coronary ostium.

Axial images, multi-planar and volume rendering reconstructions of conventional and monoenergetic images at 40 keV and 70 keV were used for the analysis (Figure 1).

Two radiologists with five and ten years of experience in cardiovascular imaging independently assessed the image quality of each dataset using a 5-point Likert scoring system (1: poor quality, not diagnostic; 2: sufficient; 3: satisfactory; 4: good; and 5: excellent). Readers were blinded to the type of dataset, and they evaluated each of the following parameters: sharpness; vascular contrast; image noise; stenosis demarcation; overall impression.

All datasets were evaluated using an initial window width/level of 900/100 HU, as recommended by the vendor; however, window settings could be subjectively adjusted by the readers. Scores were averaged and compared for conventional and monoenergetic datasets.

### 2.6. Coronary Stenosis Assessment

All CCTA datasets (i.e., conventional, VMI 40 keV, and VMI 70 keV) were reformatted using true axial and curved multiplanar reconstructions, and the degree of stenosis was semiautomatically assessed by the vendor software (Stenosis analysis, Intellispace Portal).

The degree of stenosis was expressed in percentage and the values were categorized as suggested by the SCCT grading for stenosis, in the following six groups: 0%, 1–24%, 25–49%, 50–69%, 70–99%, and 100%, as suggested by the Society of Cardiovascular Computed Tomography (SCCT) [17].

### 2.7. Statistical Analysis

Statistical analysis was performed using SPSS Statistics software (version 26.0, IBM, Armonk, NY, USA). The Shapiro–Wilk test was used to assess normality of data distribution.

Continuous variables were described as mean ± SD if normally distributed, or median (IQR) for non-normally distributed data.

To test differences between variables, analysis of variance (ANOVA) test was used for normally distributed data, while non-normal data was analyzed using the Wilcoxon matched-pairs signed rank test. A *p*-value < 0.05 was used to confirm a statistically significant difference.

For subjective image quality, inter-observer agreement was calculated with the Cohen-kappa statistic (k). The Cohen-kappa statistic was also used to evaluate the correlation among the conventional dataset and monoenergetic reconstructions for grading of stenoses.

Results were interpreted as follows: slight or poor agreement (k < 0.20), fair agreement (k = 0.20–0.40), moderate agreement (k = 0.40–0.60), good agreement (k = 0.60–0.80), and excellent agreement (k > 0.80).

To further assess coronary stenosis agreement, the Bland–Altman method was used to evaluate bias between mean differences and limits of agreement in a head-to-head comparison between conventional and 70 keV images and conventional and 40 keV images.

## 3. Results

### 3.1. Study Population

**Figure 2:**
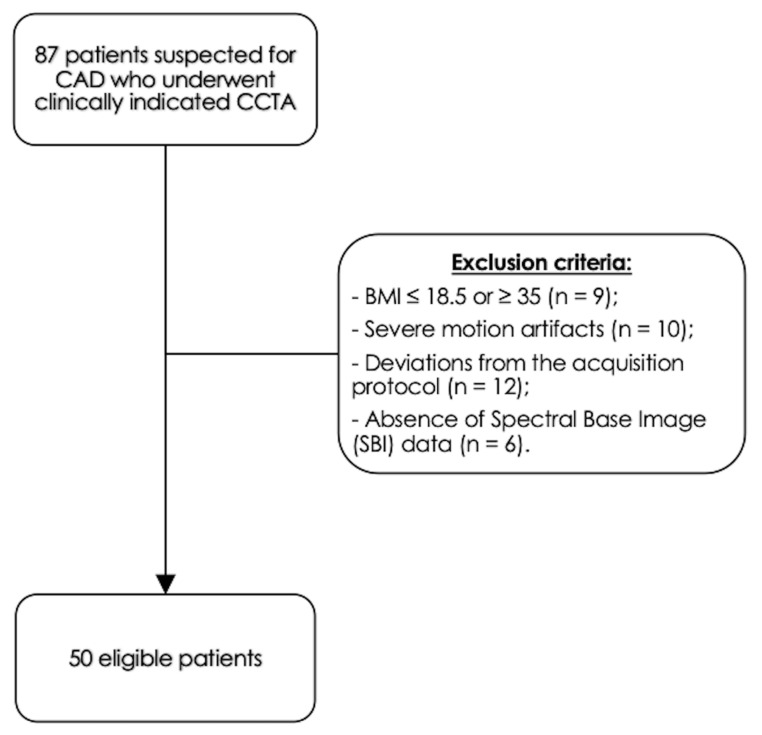
Study population.

We identified 87 consecutive eligible patients who underwent CCTA on the DLCT platform between June 2022 and December 2022 using our internal PACS. Exclusion criteria included the following: BMI ≤ 18.5 or ≥ 35 kg/m^2^, (n = 9); severe motion artifacts (n = 10); deviations from the DE-CCTA acquisition protocol due to altered tube voltage settings, kernel settings or contrast media protocol (n = 12); absence of spectral base image (SBI) data (n = 6). The final patient population consisted of 50 patients (30 males). Figure 2 displays the selection process in this study.

Image datasets from a total of 50 patients (mean age of 61.6 ± 12.3 years) were analyzed. The mean body mass index was 25.7 ± 3.6 kg/m^2^. The mean Agatston calcium score was 256.2 ± 265.4, with the presence of at least one calcified coronary plaque in 19/40 patients. The patients’ demographic characteristics are summarized in Table 1.

### 3.2. Objective Image Quality Analysis

Objective image quality analysis was performed by measurement of mean attenuation, noise, SNR, and CNR for each coronary vessel.

As for mean attenuations, distribution of the values was normal for each vessel. Attenuation progressively increased for monoenergetic reconstructions with lower keV, as normally expected. The mean attenuation of coronary arteries in conventional images (464.94 HU ± 66.39 HU) was significantly lower in comparison with 70 keV monoenergetic images (515.20 HU ± 82.85 HU, *p* = 0.03), according to literature data [18].

The 70 keV VMI images showed lower noise (8.66 HU ± 1.84 HU) than conventional images (11.58 HU ± 2.84 HU, *p* < 0.001) and 40 keV monoenergetic images (10.96 HU ± 2.91 HU, *p* < 0.001). The mean image noise of 40 keV monoenergetic reconstructions was lower than conventional images, although not significantly (*p* = 0.83). Overall, the mean values for noise constantly decreased from 40 keV towards 100 keV monoenergetic reconstructions, and the difference with conventional images became statistically significant from 50 keV VMI (9.63 HU ± 2.24 HU, *p* = 0.002).

Subsequently, SNR and CNR values were higher at lower-keV monoenergetic images and progressively decreased towards 100 keV reconstructions. In particular, all reconstructions from 70 keV to 40 keV showed significantly higher SNR (from 61.33 ± 12.46 to 154.22 ± 42.91, respectively) and CNR (from 51.45 ± 11.19 to 135.63 ± 39.38, respectively) than conventional images (all *p* < 0.001).

**Figure 3:**
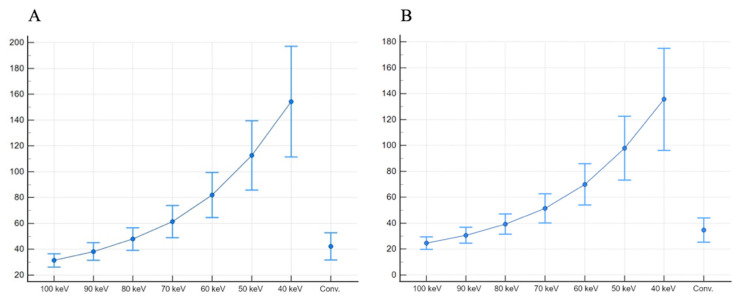
SNR (**A**) and CNR (**B**), presented as boxplots, show higher values for 40 keV monoenergetic reconstructions, when compared both with other monoenergetic reconstructions and conventional images.

Values of mean attenuation for each vessel and subcutaneous fat noise are presented in Table 2. The SNR and CNR results are presented as box plots in Figure 3.

### 3.3. Subjective Image Assessment

A five-point Likert scale was used by two experienced radiologists to rate subjective image parameters. The inter-reader agreement was good for all the parameters. The kappa values for image noise, sharpness, vascular contrast both for conventional, 70 and 40 keV monoenergetic images were all above 0.60.

The 40 keV monoenergetic images obtained the best average score for sharpness, vascular contrast, and for overall impression (all *p* < 0.001). The 70 keV monoenergetic images obtained better scores for sharpness, vascular contrast, and for overall impression compared with conventional images (all with *p* < 0.001).

The 70 keV monoenergetic images were rated as the less noisy (all *p* < 0.001). The difference in the noise scores between conventional and 40 keV monoenergetic images was not significant (*p* = 0.06), despite the 40 keV VMI obtaining higher scores.

Stenosis demarcation obtained better scores either with 70 keV and 40 keV reconstructions in comparison with conventional images (both *p* < 0.001). The difference between 40 keV and 70 keV is not significant (*p* = 0.096).

**Figure 4:**
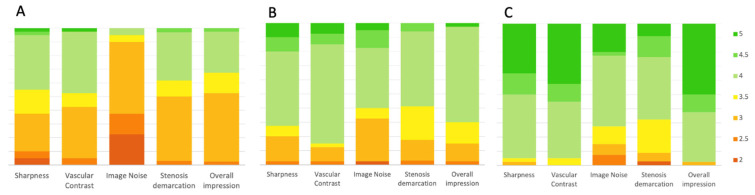
Bar plots showing results from subjective image quality. Sharpness, vascular contrast, image noise, stenosis demarcation, and overall image quality were rated by two observers. The values represent the average scores given by the two observers. The 70 keV (**B**) and 40 keV (**C**) monoenergetic reconstructions show better results if compared with conventional images (**A**).

The median of average scores with 95% confidence interval (CI) for each parameter, with the inter-reader agreement are presented in Figure 4 and in Table 3.

### 3.4. Coronary Stenosis Assessment

The grading of stenosis of coronary arteries showed excellent inter-rater agreement (overall k = 0.94 [0.91–0.97]) among conventional and monoenergetic images at 70 keV and 40 keV.

The highest inter-rater agreement was between conventional and 70 keV monoenergetic images (k = 0.97 [0.95–0.99]). Similarly, the agreement between conventional and 40 keV monoenergetic reconstruction was almost excellent (k = 0.88 [0.82–0.95]). Per-vessel categorization at different image reconstructions is shown in Table 4.

**Figure 5:**
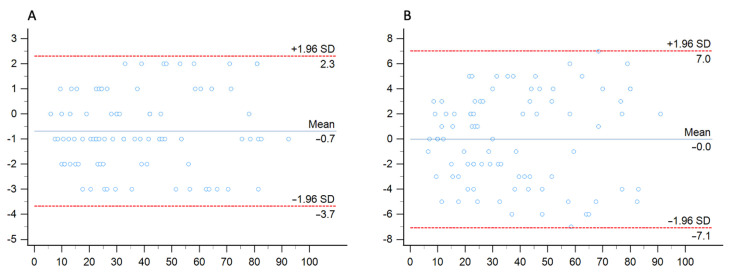
Bland–Altman analysis comparing the stenosis quantification between conventional images and 70 keV (**A**) and 40 keV (**B**) monenergetic images.

Bland–Altman analysis showed close agreement between conventional and 40 keV monoenergetic images, with a mean difference of −0.2% (−0.79–0.74) with LOA ranging between −7.06% to 7.02%. Similarly, the mean difference between conventional and 70 keV monoenergetic images was −0.7% (−1.0152–0.3641), showing smaller LOA (−3.68% to 2.30%) Figure 5.

## 4. Discussion

We compared objective and subjective image quality of monoenergetic imaging with conventional images from CCTA performed on a dual-layer spectral detector CT platform. We found that lower-keV images show the best image quality, with the highest values for contrast- and signal-to-noise ratio.

Subjective image quality parameters were also better for 40 keV monoenergetic images, and 70 keV reconstructions showed also better scores than conventional images.

This can be explained by the higher attenuation of iodine at low-keV reconstructions when the energy level approaches the iodine k-edge of 33.2 keV. This results in higher SNR and CNR values and optimal vascular contrast [19].

However, previous studies showed limitations in the use of low-keV reconstructions. First, low-keV images can determine an excessive increase of attenuation [7] leading to blooming artifacts and image quality worsening [8].

Moreover, multiple studies on different DECT platforms showed that image noise and reconstruction energy level are inversely associated in VMI. Our results did not show a significant increase of noise compared to conventional images even at the lowest keV reconstructions (40 keV). This might be partially explained by the different DECT configuration that we used to investigate VMI. In fact, the DLCT system can reduce the image noise at low energy levels due to spectral detector technology [20,21]. This translates in improved contrast enhancement at low-keV images compared with conventional imaging or allows the assumption that similar image quality can be maintained using lower volumes of contrast media [22].

Different DECT platforms, such as rapid kV-switching or dual-source scanners, perform their spectral separation on the image-domain. However, these DECT configurations have been shown to increase image noise at low-keV; despite this, limitation has been partially overcome by adopting noise-optimized algorithms that improve the image quality of the low-keV monoenergetic images [23,24,25].

Arendt et al. in their study found that 40 keV noise-optimized (VMI+) obtained by a dual-source platform significantly improve both objective and subjective image quality in CCTA examinations [9].

Our results obtained on a DLCT system showed that 40 keV images have the best SNR and CNR with similar noise levels to conventional images. Similar, to current literature, 70 keV images showed improved image noise compared to conventional and 40 keV images.

Nevertheless, the use of monoenergetic reconstructions did not alter the coronary stenosis quantification, keeping good the agreement among conventional, 70 keV and 40 keV monoenergetic images. However, optimal image quality needed manual adjustment of window settings by the readers to avoid blooming of the vessel, which might be dependent on the experience of the reader.

In clinical practice this may translate in better image quality, achievable by means of DLCT monoenergetic imaging without compromising the coronary lumen assessment, and without increasing the patient’s radiation exposure [5]. On the other hand, the higher CNR of low keV images may also allow CCTA with lower volumes of contrast media to be performed [26].

Our study has several limitations. First, it is a single center study with a retrospective design. Second, our sample size is limited and larger studies with prospective design would be more favorable. Third, we did not perform a diagnostic accuracy analysis to test the performance of the VMI datasets for grading coronary stenosis, since coronary angiograms were not available for the majority of patients. Fourth, we did not test whether the improved image quality of VMI datasets may significantly affect the diagnostic performance of readers with different years of experience in cardiovascular imaging.

## Figures and Tables

**Table 1:** Demographic characteristics of study population.

Characteristics	Both Sexes	Men	Women
**Number (%)**	50 (100%)	30 (60.0%)	20 (40.0%)
**Age (years): Mean (SD)**	61.6 (12.3)	62.2 (11.7)	60.6 (13.5)
**BMI: Mean kg/m^2^ (SD)** **18.5–24.9: number (%)** **25–29.9: number (%)** **30–34.9: number (%)**	25.7 (3.6) 25 (50%) 20 (40%) 5 (10%)	26.3 (3.8) 12 (40.0%) 12 (40%) 4 (13.3%)	24.9 (3.2) 11 (55%) 8 (40%) 1 (5%)
**Calcium Score (Agatston): Mean (SD)**	256.2 (265.4)	309.3 (290.4)	180.6 (210.0)
**CAC-DRS:** **A: median (IQR)** **N: median (IQR)**	2 (0–3) 1 (0–3)	3 (0–3) 1.5 (0–3)	1 (0–3) 0.5 (0–1.5)

**Table 2:** Data are presented as mean (HU) ± SD. Attenuation, signal-to-noise ratio (SNR) and contrast-to-noise ratio (CNR) are reported for ascending aorta, left main coronary artery (L.main), left anterior descending artery (LAD), left circumflex coronary artery (LCx), right coronary artery (RCA). The standard deviation of subcutaneous fat attenuation is reported for image noise.

	Conventional	VMI 100 keV	VMI 90 keV	VMI 80 keV	VMI 70 keV	VMI 60 keV	VMI 50 keV	VMI 40 keV
**Aorta** **Attenuation (HU)** **SNR** **CNR**	443.93 ± 59.05 40.49 ± 10.50 32.96 ± 9.21	241.3 ± 33.49 29.73 ± 5.72 22.94 ± 5.15	298.18 ± 44.21 36.84 ± 7.48 29.33 ± 6.67	381.51 ± 59.37 46.78 ± 9.37 38.23 ± 8.47	521.14 ± 83.51 60.94 ± 12.75 51.05 ± 11.68	717.33 ± 120.84 82.33 ± 16.86 70.27 ± 15.80	1064.17 ± 182.13 114.42 ± 25.48 99.74 ± 23.98	1673.69 ± 281.09 157.42 ± 40.46 138.84 ± 37.79
**L.main** **Attenuation (HU)** **SNR** **CNR**	451.28 ± 62.68 40.68 ± 10.73 33.14 ± 9.51	251.47 ± 40.21 30.89 ± 5.84 24.10 ± 5.41	306.01 ± 48.62 37.73 ± 7.42 30.22 ± 6.66	387.60 ± 62.96 47.47 ± 9.27 38.92 ± 8.35	510.30 ± 81.94 60.89 ± 12.83 51.00 ± 11.63	717.67 ± 121.15 81.99 ± 18.03 69.93 ± 16.70	1044.98 ± 182.33 113.02 ± 27.99 98.34 ± 26.07	1605.15 ± 284.26 155.18 ± 44.57 136.59 ± 41.41
**LAD** **Attenuation (HU)** **SNR** **CNR**	464.61 ± 76.37 42.42 ± 11.75 34.88 ± 10.57	254.02 ± 51.29 31.11 ± 6.70 24.33 ± 6.56	307.36 ± 58.85 37.76 ± 7.92 30.25 ± 7.48	387.17 ± 72.2 47.39 ± 9.84 38.84 ± 9.13	510.47 ± 93.91 60.79 ± 13.53 50.90 ± 12.43	704.19 ± 129.29 81.09 ± 18.69 69.03 ± 17.36	1031.51 ± 186.08 111.45 ± 27.91 96.67 ± 25.91	1578.37 ± 288.46 152.58 ± 44.26 133.99 ± 40.92
**LCx** **Attenuation (HU)** **SNR** **CNR**	474.79 ± 77.09 43.08 ± 10.96 35.55 ± 9.85	269.73 ± 45.10 32.90 ± 5.04 26.11 ± 4.96	324.66 ± 53.73 39.81 ± 6.90 32.31 ± 6.32	404.49 ± 68.73 49.33 ± 8.90 40.78 ± 8.07	526.31 ± 92.64 62.55 ± 12.68 52.66 ± 11.48	720.30 ± 133.11 82.74 ± 17.96 70.68 ± 16.43	1044.94 ± 197.95 112.59 ± 27.56 97.90 ± 25.42	1591.35 ± 310.15 153.43 ± 44.73 134.84 ± 41.21
**RCA** **Attenuation (HU)** **SNR** **CNR**	474.57 ± 83.68 42.90 ± 10.84 35.37 ± 9.87	251.12 ± 42.64 30.62 ± 4.89 23.83 ± 4.57	307.58 ± 52.30 37.74 ± 7.31 30.23 ± 6.52	388.67 ± 65.68 47.44 ± 9.26 38.89 ± 8.25	513.7 ± 86.51 61.11 ± 13.08 51.22 ± 11.74	714.62 ± 118.83 82.17 ± 18.16 70.11 ± 16.42	1049.19 ± 174.94 113.24 ± 27.56 98.55 ± 25.27	1613.62 ± 258.94 155.71 ± 42.23 137.11 ± 39.56
**Subcutaneous fat (SD)**	11.53 ± 2.84	8.32 ± 1.47	8.34 ± 1.59	8.39 ± 1.65	8.66 ± 1.84	8.97 ± 1.96	9.63 ± 2.24	10.96 ± 2.91

**Table 3:** Comparison between conventional and monoenergetic images. 70 and 40 keV monoenergetic reconstructions show better subjective image parameters in comparison with conventional images.

Score and Interobserver agreement (k)			
	Conventional images	VMI 70 keV	VMI 40 keV
**Image sharpness**	3.5 (3–4) *k = 0.67 [0.47–0.87]*	4 (3.5–4) *k = 0.64 [0.44–0.84]*	4.25 (4–5) *k = 0.70 [0.49–0.91]*
**Image noise**	3 (2.5–3) *k = 0.64 [0.42–0.87]*	4 (3–4) *k = 0.66 [0.46–0.86]*	4 (3.5–4) *k = 0.64 [0.46–0.82]*
**Vascular contrast**	3.5 (3–4) *k = 0.68 [0.47–0.88]*	4 (4–4) *k = 0.67 [0.47–0.88]*	4.5 (4–5) *k = 0.68 [0.49–0.89]*
**Stenosis demarcation**	3 (3–4) *k = 0.81 [0.70–0.93]*	3.5 (3–4) *k = 0.76 [0.61–0.91]*	4 (3.5–4) *k = 0.73 [0.58–0.89]*
**Overall impression**	3 (3–4) *k = 0.62 [0.41–0.84]*	4 (3.5–4) *k = 0.61 [0.36–0.86]*	4.75 (4–5) *k = 0.77 [0.57–0.97]*
**Statistical comparison**			
	**Conventional vs.** **VMI 70 keV**	**Conventional vs.** **VMI 40 keV**	**VMI 70 keV vs.** **VMI 40 keV**
**Image sharpness**	*p* < 0.001	*p* < 0.001	*p* < 0.001
**Image noise**	*p* < 0.001	*p* = 0.061	*p* < 0.001
**Vascular contrast**	*p* < 0.001	*p* < 0.001	*p* < 0.001
**Stenosis demarcation**	*p* = 0.018	*p* < 0.001	*p* = 0.096
**Overall impression**	*p* < 0.001	*p* < 0.001	*p* < 0.001

**Table 4:** Per-vessel analysis of coronary stenosis using conventional, 70 keV and 40 keV monoenergetic imaging by means of semi-automated analysis. L. Main: Left main coronary artery; LAD: Left anterior descendent coronary artery; LCx: left circumflex coronary artery; RCA: Right coronary artery.

Artery	Conv.	VMI 70 keV	VMI 40 keV
**L. Main** - **0%** - **1–24%** - **25–49%** - **50–69%** - **70–99%** - **100%**	33 3 3 1 0 0	33 4 2 1 0 0	33 4 2 1 0 0
**LAD** - **0%** - **1–24%** - **25–49%** - **50–69%** - **70–99%** - **100%**	8 11 9 7 5 0	8 10 10 7 5 0	8 12 8 6 6 0
**LCx** - **0%** - **1–24%** - **25–49%** - **50–69%** - **70–99%** - **100%**	18 9 7 2 4 0	18 8 8 1 5 0	18 8 8 4 2 0
**RCA** - **0%** - **1–24%** - **25–49%** - **50–69%** - **70–99%** - **100%**	14 9 12 3 2 0	14 9 12 3 2 0	14 10 9 5 2 0
